# Identifying determinants for the seropositive rate of schistosomiasis in Hunan province, China: A multi-scale geographically weighted regression model

**DOI:** 10.1371/journal.pntd.0011466

**Published:** 2023-07-13

**Authors:** Yixin Tong, Ling Tang, Meng Xia, Guangping Li, Benjiao Hu, Junhui Huang, Jiamin Wang, Honglin Jiang, Jiangfan Yin, Ning Xu, Yue Chen, Qingwu Jiang, Jie Zhou, Yibiao Zhou

**Affiliations:** 1 Fudan University School of Public Health, Shanghai, China; 2 Key Laboratory of Public Health Safety, Fudan University, Ministry of Education, Shanghai, China; 3 Fudan University Center for Tropical Disease Research, Shanghai, China; 4 Hunan Institute for Schistosomiasis Control, Yueyang, China; 5 School of Epidemiology and Public Health, Faculty of Medicine, University of Ottawa, Ottawa, Canada; Federal University of Agriculture Abeokuta, NIGERIA

## Abstract

**Background:**

Schistosomiasis is of great public health concern with a wide distribution and multiple determinants. Due to the advances in schistosomiasis elimination and the need for precision prevention and control, identifying determinants at a fine scale is urgent and necessary, especially for resource deployment in practice. Our study aimed to identify the determinants for the seropositive rate of schistosomiasis at the village level and to explore their spatial variations in local space.

**Methodology:**

The seropositive rates of schistosomiasis were collected from 1714 villages or communities in Human Province, and six spatial regression models including ordinary least squares (OLS), spatial lag model (SLM), spatial error model (SEM), geographically weighted regression (GWR), robust GWR (RGWR) and multiscale GWR (MGWR) were used to fit the data.

**Principal/Findings:**

MGWR was the best-fitting model (R^2^: 0.821, AICc:2727.092). Overall, the nearest distance from the river had the highest mean negative correlation, followed by proportion of households using well water and the annual average daytime surface temperature. The proportions of unmodified toilets showed the highest mean positive correlation, followed by the snail infested area, and the number of cattle. In spatial variability, the regression coefficients for the nearest distance from the river, annual average daytime surface temperature and the proportion of unmodified toilets were significant in all villages or communities and varied little in local space. The other significant determinants differed substantially in local space and had significance ratios ranging from 41% to 70%, including the number of cattle, the snail infested area and the proportion of households using well water.

**Conclusions/Significance:**

Our study shows that MGWR was well performed for the spatial variability of schistosomiasis in Hunan province. The spatial variability was different for different determinants. The findings for the determinants for the seropositive rate and mapped variability for some key determinants at the village level can be used for developing precision intervention measure for schistosomiasis control.

## Author summary

The elimination of schistosomiasis depends critically on the identification of a set of determinants. A site-specific analysis of the determinants allows organizers to adjust and clarify the focus areas for the next round. This analysis, based on anecdotal evidence and investigations specific to a narrow geographic area at a fine scale, would enhance our understanding of the determinants. Especially for areas where elimination goals are to be achieved, local spatial variation in determinants is worth exploring in depth.

In this analysis we included the seropositive rates of schistosomiasis from 1714 villages or communities and established six spatial regression models. Benefiting from determinants with different bandwidths, MGWR was good at simulating schistosomiasis. We also explored the spatial variability of multiple determinants and mapped variability for some key determinants at the village level. We hope the findings will be used for developing precision intervention measure for schistosomiasis control.

## 1. Introduction

Schistosomiasis, caused by infecting with schistosomes, is a parasitic disease with a global distribution of 78 countries and regions, located in Asia, Africa and Latin America [1]. Over the past decade, schistosomiasis has claimed between 1.5 and 2.5 million years lived with disability (YLDs) per year [2,3]. It is estimated that over 240 million people worldwide are affected by schistosomiasis, and the vast majority of them live in low- and middle-income countries (LMICs) [4,5]. The disease is significantly correlated with both poverty and poor public health, which highlights the importance of social infrastructure and public health measures [6]. Despite the widespread implementation of targeted preventive chemotherapy, schistosomiasis remains highly prevalent in many endemic areas [7] and has been receiving an increasing attention in recent years, partly due to its link to increased susceptibility HIV-1 infection [8].

In China, schistosomiasis is one of the highest priorities identified by the government for infectious disease control [9], but there are new challenges emerged in recent years [10]. Due to the low parasite load after treatment, the Kato Katz Method (KKM) is not sensitive enough to determine the status of schistosomiasis in hotspots [11,12]. Misdiagnosis caused by inaccurate parasitological tests is also common in the context of low infection rates as a result of prevention and control measures [13,14], and more accurate methods are needed [11,15]. Indirect hemagglutination screening (IHA) is widely used as a complementary or alternative diagnostic method due to its high sensitivity and low-cost [11,15]. Especially in areas with numerous epidemiological hotspots, underdeveloped economies and dense populations, it is important to use the IHA to identify all possible schistosome infections.

The transmission of schistosomiasis is complex and heterogeneous and is affected by many factors, including environmental and demographic factors [16,17]. Cattle are also important animal factors and 90% of the transmission of schistosomiasis in China occurs in cattle [18–20]. In addition, behavior factors are another crucial element. The WHO recommendations for 2022 highlight the need and urgency for behavioral change interventions to halt the progression of schistosomiasis in endemic areas [21]. Furthermore, Lo et al. noted the analysis of data at the village level is also necessary, which would help monitor and track effective coverage of these persistent hot spots in behavior intervention efforts, and to prioritize them for additional intervention [21].To develop effective intervention measures, especially for the persistent hot spots, it is important to identify the determinants of schistosomiasis transmission at fine scales [16,22] such as at the village level [21].

Spatial models, such as global and local regression models, can be used to examine the geographical determinants [16,23] and to describe long-term dynamics of diseases [24]. Clearly defining the exact areas and associations for determinants help further tailor efficient treatments to local conditions. This study aimed to identify determinants for the seropositive rate of schistosomiasis at the village level and to explore spatial variation in local space, so as to provide guidelines for developing precision intervention measure for schistosomiasis control.

## 2. Materials and methods

### 2.1. Study area

The study area is situated in Hunan Province, China, between 24°37′ and 30°08′ N north latitude and 108°47′ and 114°16′ E east longitude [25]. With 24,986 estimated schistosomiasis cases in 2018, Hunan Province is the main schistosomiasis endemic region in China [26,27]. The endemic areas in Hunan are in 41 surrounding counties of the Dongting Lake.

### 2.2. Data collection and preparation

In autumn 2020, a sampling survey of schistosomiasis was conducted in Hunan Province. This survey included all villages or communities that had not reached the goal of transmission interruption as well as some villages or communities that had reached the goal of transmission interruption and disease elimination but required to be surveyed in rotation within three years and five years, respectively. Indirect hemagglutination screening (IHA) was applied as a diagnostic tool for schistosomiasis infection in this survey. We calculated the seropositive rate as the number of IHA positives divided by the total number of IHA testers at the village level. The village levels referred to the village or community as the basic unit. Further, seropositive rates were joined to the administrative boundary shapefile of villages or communities in ArcGIS (Esri, Redlands, CA, USA) [26] At the same time, determinants related to schistosomiasis, such as number of populations, fishermen, cattle, and sheep, the snail infested area, proportions of households using well water, unmodified toilet, and biogas digester, were collected at the village level.

### 2.3. Determinant factors

We compiled socioeconomic, demographic, environmental, animal and behavioral factors from this field survey as well as public databases such as gross domestic product, surface temperature and climate (S1 Table). The nearest distance from the river was calculated based on the map of rivers in China in ArcGIS. We unified the projection coordinate system and extracted them to the extent of Hunan Province. Then, all factors were collated and matched according to coordinates in ArcGIS at the village level and normalized to eliminate the influence from their different dimensions and units [28].

We used stepwise regression to explore the relationship between these factors and the seropositive rate. To avoid the effect of multicollinearity, we only kept the statistically significant factors (*P*<0.05) with the variance inflation factor (VIF) less than 5 [28,29], and 8 factors were included in the final spatial models (Table 1). The statistical descriptions for the selected factors before normalizing were summarized in Table 2.

**Table 1:** The descriptions, formats and sources of the factors in global and local spatial regression models.

Factors	Descriptions	Year	Formats	Sources
GDP	Gross domestic product	2020	Raster (1km)	https://www.resdc.cn
Daylst	Annual average daytime surface temperature	2020	Raster (1km)	https://www.resdc.cn
WIN	Annual average wind speed	2020	Raster (1km)	https://www.resdc.cn
Cattle	Number of cattle in a village or community	2020	Shp	Survey
Snails	Snail infested area in a village or community	2020	Shp	Survey
Wellwater	Proportion of households using well water in total households for a village or community	2020	Shp	Survey
UToilets	Proportion of unreformed toilets in the total toilets for a village or community	2020	Shp	Survey
Riverdis	The nearest distance from the river	2020	Shp	https://www.cehui8.com/

**Table 2:** Statistical descriptions for the selected factors.

Factors	Units	Max	Min	Mean	P25	P75
GDP	10^4^yuan/km^2^	120836.000	450.000	5158.267	1548.500	2608.750
Daylst	°C	27.498	19.617	22.799	22.199	23.348
WIN	m/s	2.083	1.433	1.901	1.814	1.999
Cattle	-	602.000	0.000	11.751	0.000	0.000
Snails	m^2^	129662400.000	0.000	968435.975	0.000	117375.000
Wellwater	%	2.099	0.000	0.165	0.000	0.000
UToilets	%	2.762	0.000	0.138	0.000	0.020
Riverdis	m	87990.404	0.653	12843.079	1766.403	14331.834

### 2.4. Global spatial analysis

#### 2.4.1. Ordinary least squares (OLS)

OLS is a global spatial regression method [23], and assumes that dependent variables are spatially independent of each other without spatial dependence and is generally defined as [30]:

$$y_{i}=\beta_{0}+{\beta x}_{i}+\varepsilon_{i}$$ (1)

#### 2.4.2. Spatial lag model (SLM)

SLM assumes an interdependence between the dependent variable and explanatory variables. The addition of a spatially lagged dependent variable to the regression model is used to account for inherent spatial interactions and dependencies [31,32]. SLM is generally defined as [33]:

$$y_{i}=\beta_{0}+{{\rho w}_{i}y}_{i}+\beta x_{i}+\varepsilon_{i}$$ (2)

where *ρ* is the spatial autoregressive parameter and *w*_*i*_ is a spatial weights matrix. The spillover effects of nearby sites are described by both the spatial lag-dependent variable and the weight matrix [34]. Lee has proved that OLS is a consistent estimator for a spatial lag specification based on the group-wise weights [35].

#### 2.4.3. Spatial error model (SEM)

SEM also assumes an interdependence between the dependent variable and explanatory variables. It works through the error term to describe the spatial dependence, which is generally defined as [33]:

$$y_{i}=\beta_{0}+\lambda w_{i}\xi_{i}+{\beta x}_{i}+\varepsilon_{i}$$ (3)

where *ξ*_*i*_ is the spatial component of the error and *λ* is the level of correlation between these components. SEM is also one of the variants of OLS, incorporating spatial weights and measuring spatial dependence with an error term.

### 2.5. Local spatial analysis

#### 2.5.1. Geographically weighted regression (GWR) and robust GWR (RGWR)

Based on the development of the general regression model, GWR allows for spatial variation in the relationship between explanatory variables and dependent variables, as known as “parameters to vary spatially” [36]. GWR reveals natural associations between variables by assigning higher weights to observations near sample points [37]. Basic GWR is generally defined as:

$$yi=\beta_{0}(u_{i},v_{i})+\sum_{j=1}^{m}\beta_{j}(u_{i},v_{i})x_{ij}+\varepsilon_{i}$$ (4)

where *β*_0_(*u*_*i*_, *v*_*i*_) is the intercept; *β*_j_(*u*_*i*_, *v*_*i*_) is the value of the continuous function at point (*u*_*i*_, *v*_*i*_); *x*_*ij*_ is the value of the *j*th explanatory variable at point *i*; and *ε*_*i*_ is a random error term.

The effect of outliers is weakened in RGWR [38]. Sample data with large external studentized residuals are first removed from the initial GWR fit, and then the filtered data are refitted using the basic GWR [39].

#### 2.5.2. Multiscale GWR (MGWR)

MGWR is an extension of GWR that allows the scale of all relationships are not fixed over space [40]. MGWR matches each explanatory variable to its unique bandwidth. Differences in bandwidth represent differences in spatial scale, and by capturing the effects of scale in spatial processes, MGWR can capture spatial heterogeneity more accurately [41]. MGWR is generally defined as [40]:

$$y_{i}=\sum_{j=1}^{m}\beta_{0}x_{ij}+\sum_{j=1}^{m}\beta_{bwj}(u_{i},v_{i})x_{ij}+\varepsilon_{i}$$ (5)

where *bwj* is the bandwidth used by the *j*th variable regression coefficient. MGWR is calibrated using a back-fitting algorithm and can be reformulated as a generalized additive model in practice [23,40].

### 2.6. Model development

To examine the relationship between determinants and the seropositive rate, we used six spatial models including three global models (ordinary least squares (OLS), spatial lag model (SLM), spatial error model (SEM)) and three local models (geographically weighted regression (GWR), robust GWR (RGWR) and multiscale GWR (MGWR)). For approaching normality before constructing these regression models, the seropositive rate values were boxcox-transformed after adding one percent overall [42]. Global spatial models were conducted in GeoDa 1.14 (Anselin, Chicago, IL, USA) and the first-order Queens’ contiguity was used to form the weight matrix. Local spatial models were developed by an open-source platform (https://sgsup.asu.edu/sparc/mgwr) and *GWmodel* packages in R 4.1.0 (R Development Core Team, 2021). We used the corrected Akaike information criterion (AICc) to optimize the bandwidth and selected the fixed Gaussian kernel [43,44]. The model performances were evaluated by calculating the coefficient of determination (R^2^) and AICc [44,45]. Better model performance is evidenced by a higher R^2^ value and a lower AICc value.

## 3. Results

**Fig 1:**
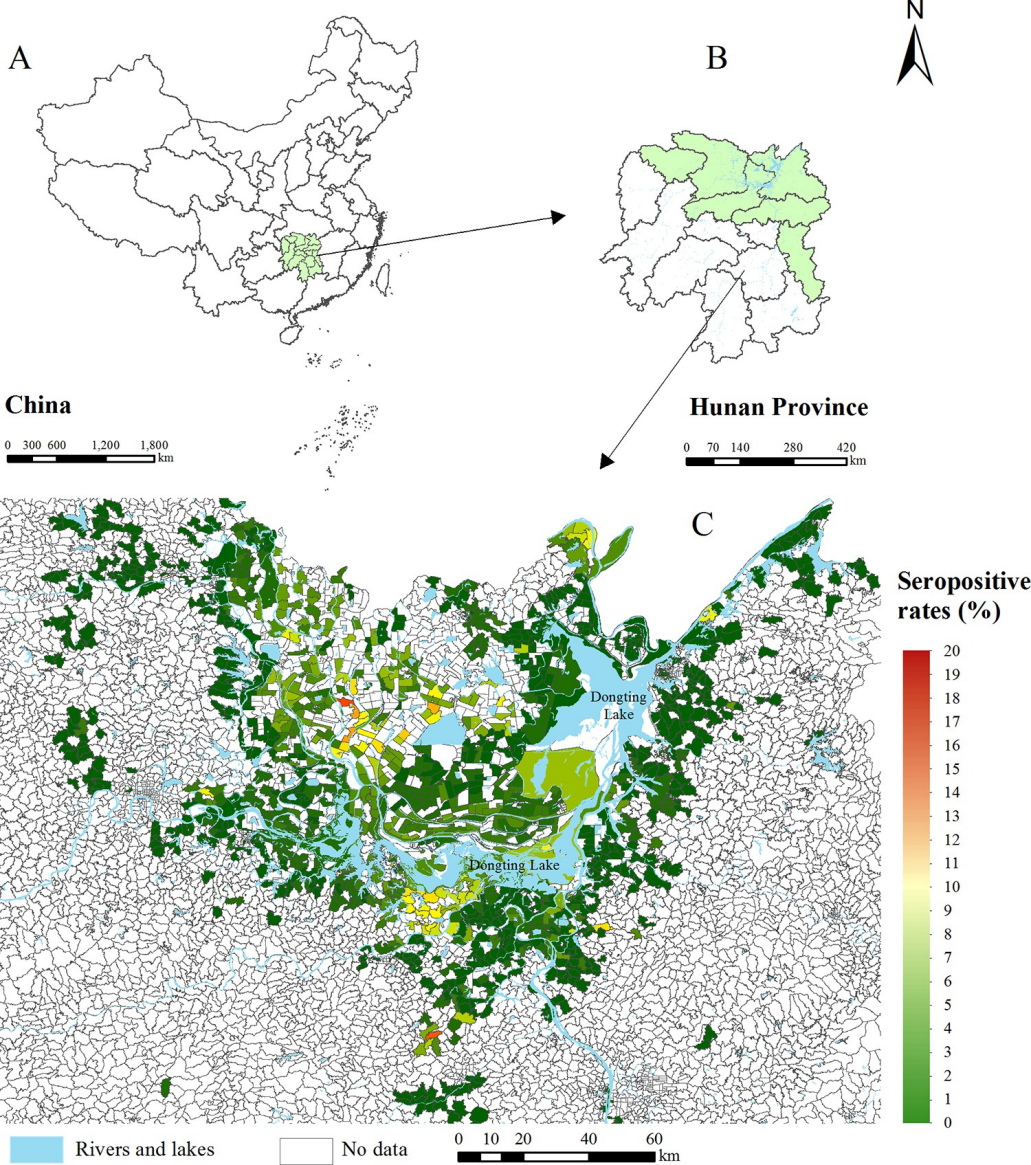
The seropositive rates of schistosomiasis in Hunan province. (A) China. (B) Hunan province. (C) Study area. The source of the basemap shapefile was from the open access platform: National Platform for Common Geospatial Information Services (www.tianditu.gov.cn).

A total of 1718 administrative villages or communities were included in our study and 32.4% (558/1718) of them had a seropositive rate of 0. Of 1160 villages or communities that had a seropositive rate greater than 0, 427 had a seropositive rate less than 1.0%, 574 had a rate between 1.0% and 4.9%, 125 had a rate between 5.0% and 9.9%, 30 had a rate between 10.0% and 19.9% and 4 had a rate between 20.0% and 60.0%. Considering the effect of outliers on the models, we removed the 4 sites with a seropositive rate of 20.0% or more (20.2%, 35.5%, 48.0% and 54.1%), and 1714 villages or communities were included in modeling. Fig 1 shows the distribution of the seropositive rates of 1714 villages or communities. The sites with a high seropositive rate were mainly distributed in the northern and southern sides of Dongting Lake.

Table 3 shows that all the factors were closely related to the seropositive rate in the OLS regression model (*P*<0.05). However, the OLS model had the poorest fitness with a low confidence in the coefficients of factors. The Jarque-Bera test scores and White’s test indicated that the errors were not normally distributed and heteroskedasticity existed (*P*<0.001). Significant Moran I (error) scores indicated the strong spatial autocorrelation among the residuals (*P*<0.001). The above effects clarified the necessity to apply other global and local spatial models in our study.

**Table 3:** Performance of global spatial regression models and their regression coefficients for study factors.

	OLS			SLM			SEM		
R^2^	0.168			0.683			0.683		
AIC	1928.310			558.747			579.088		
**Factors**	**Coefficient**	**t-value**	***P***	**Coefficient**	**z-value**	***P***	**Coefficient**	**z-value**	***P***
CONSTANT	0.503	3.015	0.003	0.119	1.148	0.251	0.676	4.693	<0.001
GDP	-0.962	-10.643	<0.001	-0.188	-3.294	<0.001	-0.192	-1.602	0.109
Daylst	-0.678	-3.703	<0.001	-0.277	-2.444	0.015	-0.327	-2.403	0.016
WIN	0.724	5.666	<0.001	0.236	2.978	0.003	0.146	1.372	0.170
Cattle	0.906	6.229	<0.001	0.393	4.382	<0.001	0.342	3.444	<0.001
Snails	0.842	3.284	0.001	0.457	2.890	0.004	0.368	2.329	0.020
Wellwater	-0.474	-7.991	<0.001	-0.160	-4.266	0.000	-0.299	-4.919	<0.001
UToilets	0.203	2.134	0.033	0.127	2.155	0.031	0.165	1.781	0.075
Riverdis	-0.418	-6.696	<0.001	-0.067	-1.696	0.090	-0.249	-1.524	0.128
ρ/λ*	-	-	-	0.813	49.562	<0.001	0.835	52.655	<0.001

Notes: * *ρ*: spatial lag term; *λ*: the error terms.

The significant Lagrange multiplier lag (LM-lag) and Lagrange multiplier error (LM-err) statistics (*P*<0.001) indicated the presence of both spatial lag and spatial error effects. In contrast, SLM and SEM performed much better than OLS, with higher values of R^2^ and lower AIC. In SLM, all the factors except for Riverdis were correlated with the seropositive rate, while all the factors except for GDP, WIN, UToilets and Riverdis were correlated with the seropositive rate in SEM. The absolute values of the regression coefficients of the factors ranked differently in the SLM and SEM (Table 3).

Table 4 shows the performance of local spatial models. R^2^ for both the GWR and RGWR were 0.792, or 79.2% of the total variation of the seropositive rate can be explained by the study factors (Table 4). RGWR excluded the effect of outliers over space. The consistency in model performance suggests that the outlier effect was not important in our study. MGWR is the best fit model with the highest R^2^ of 0.821 and the lowest AICc.

**Table 4:** Performance of local spatial regression models.

	GWR	Robust-GWR	MGWR
R^2^	0.792	0.792	0.821
AICc	3015.349	3015.349	2727.092

MGWR was used to further explore the relationship between determinants and the seropositive rate for each local space. For each factor, then we calculated the proportion of villages or communities with the significant coefficient in all villages or communities (Table 5). In the MGWR, 41% to 100% of villages or communities had significant coefficients for Daylst, Cattle, Snails, Wellwater, UToilets and Riverdis, but 0% for GDP and WIN.

**Table 5:** The regression coefficients for factors with a non-zero significance ratio in MGWR and the proportion of villages or communities with a significant regression coefficient of each study factor.

Factors	Mean	Min	Max	Significance (%)
Intercept	0.029	-1.564	2.285	58
Daylst	-0.054	-0.054	-0.053	100
Cattle	0.042	-0.022	0.078	70
Snails	0.050	0.007	0.205	41
Wellwater	-0.135	-3.052	0.571	41
UToilets	0.054	0.054	0.055	100
Riverdis	-0.513	-0.513	-0.513	100

Table 5 also shows the regression coefficients for determinants with non-zero significance ratios in MGWR. Overall, Riverdis had the highest mean negative correlation, followed by Wellwater and Daylst. UToilets had the highest mean positive correlation, followed by Snails and Cattle.

In spatial variability, the regression coefficients for Daylst, UToilets and Riverdis were significant in all villages or communities and the differences for each of these factors were very small in local space. However, other variables differed a lot in local space and had significance ratios ranging from 41% to 70%.

**Fig 2:**
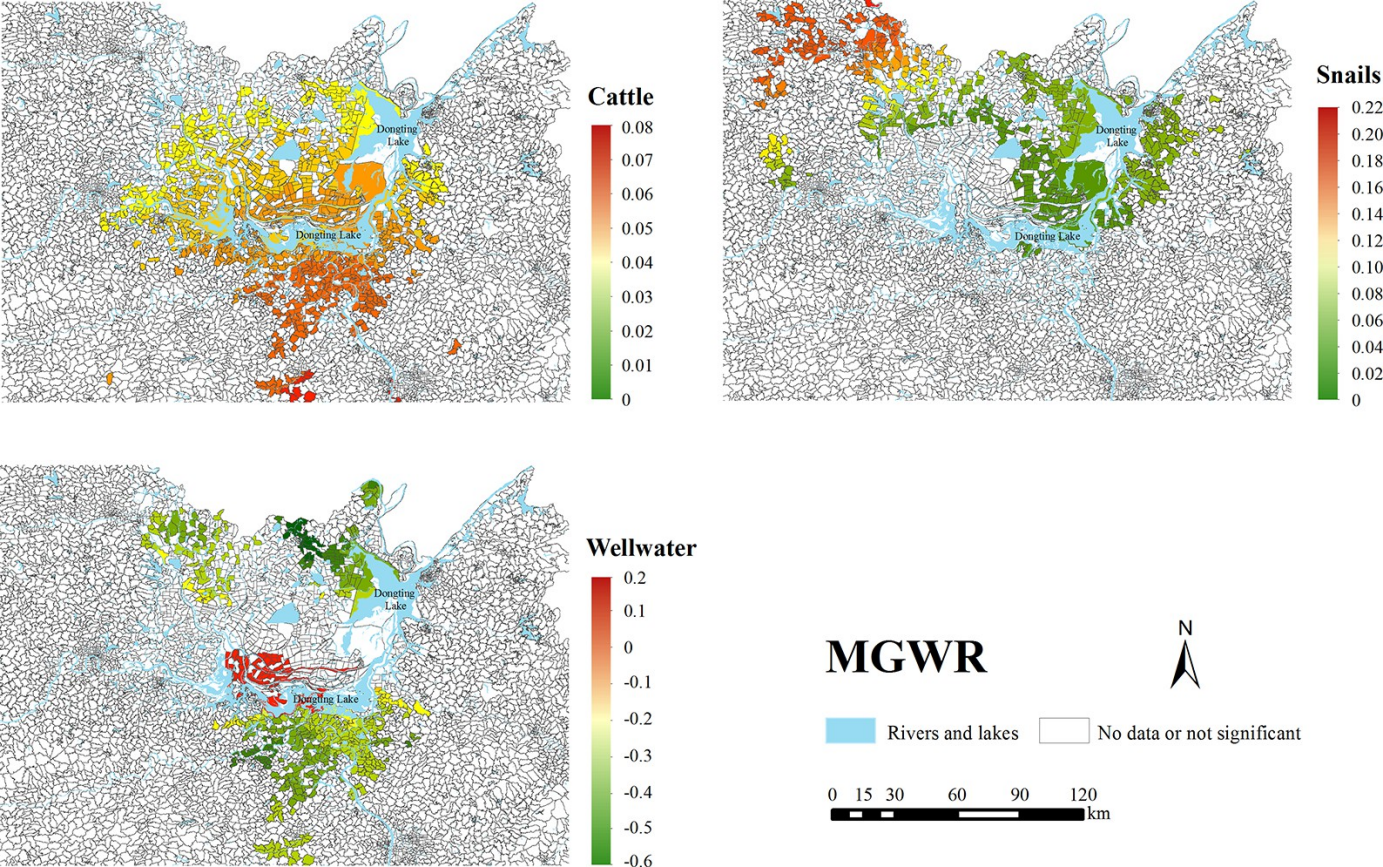
Distributions of regression coefficients for the three factors that differed substantially in space in MGWR. The source of the basemap shapefile was from the open access platform: National Platform for Common Geospatial Information Services (www.tianditu.gov.cn).

Fig 2 shows significant regression coefficients for the study factors that differed substantially in local space in MGWR. Cattle displayed a positive correlation distribution in villages or communities close to Dongting Lake, namely the closer to the lower reaches of the lake, the higher the correlation for Cattle. The regression coefficient for Cattle was not statistically insignificant for the villages or communities far from the lake. For Snails, the significant regression coefficients were concentrated in the north-western side of Dongting Lake. For Wellwater, there was an overall negative correlation, and only a few villages surroundings the lake were anomalously positively correlated.

**Fig 3:**
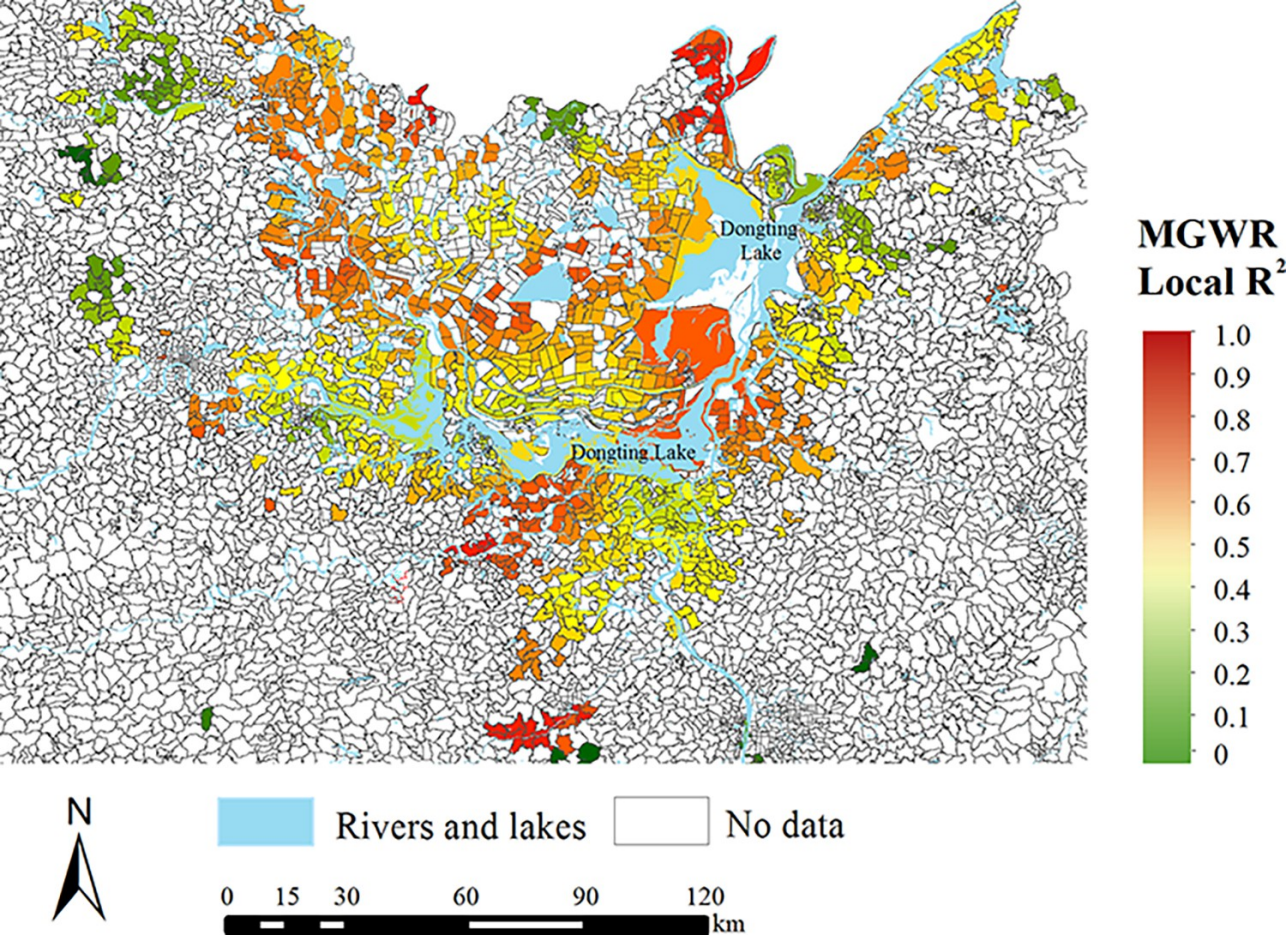
The spatial distribution of local R^2^ in MGWR for identifying the determinants of the seropositive rate of schistosomiasis. The source of the basemap shapefile was from the open access platform: National Platform for Common Geospatial Information Services (www.tianditu.gov.cn).

Fig 3 illustrates the spatial distribution of local R^2^ in MGWR. The local R^2^ in areas surrounding the Dongting Lake were generally high, indicating a good fit in MGWR. On the contrary, some scattered areas away from the Dongting Lake showed a low local R^2^ with a poor model performance. The highest local R^2^ was 0.99, with 59.1% (1013/1714) of the local R^2^ above 0.5 and 28.4% (487/1714) of the local R^2^ above 0.8.

**Fig 4:**
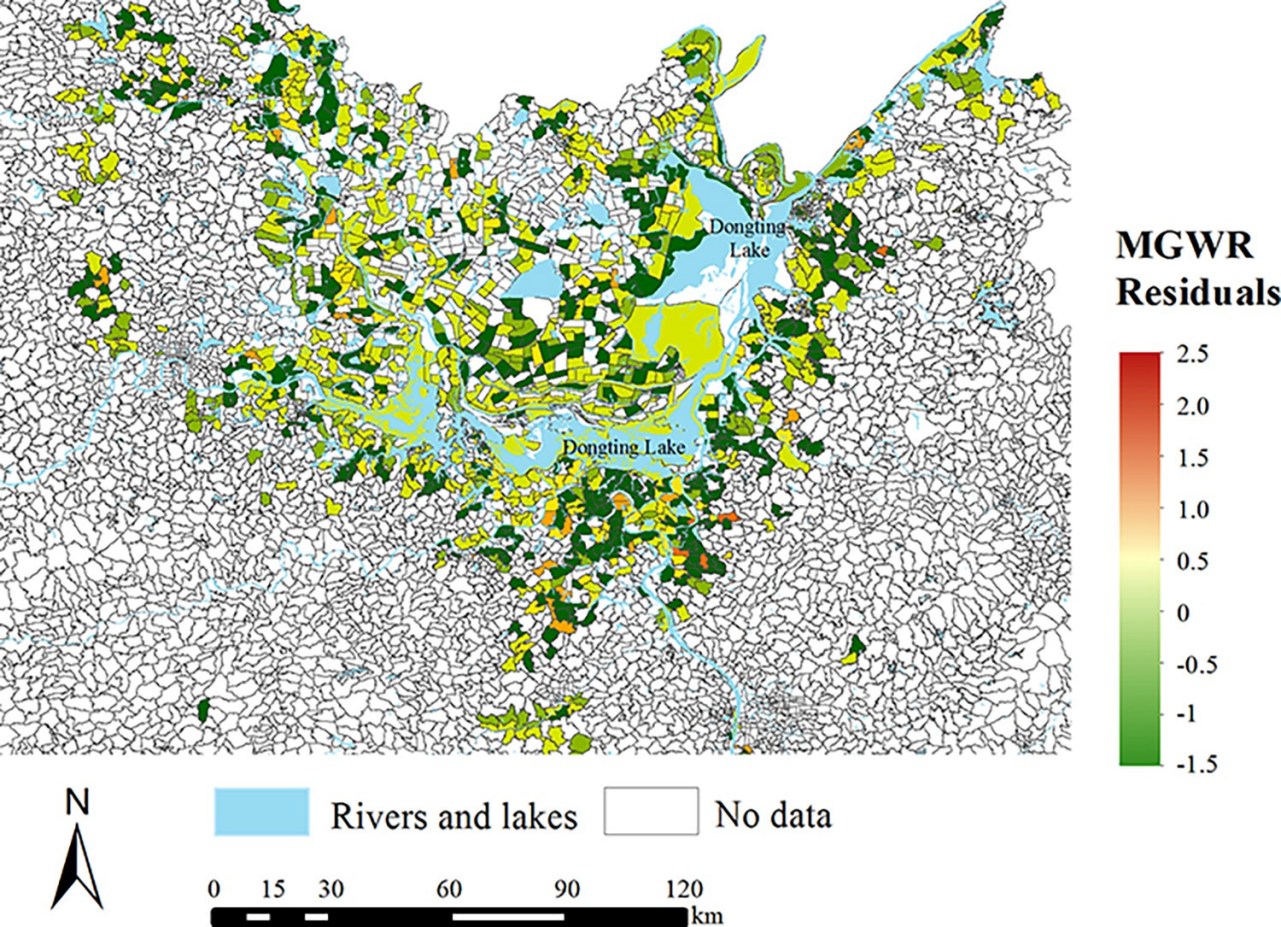
The residuals between the observed and predicted values of the seropositive rate of schistosomiasis in MGWR. The source of the basemap shapefile was from the open access platform: National Platform for Common Geospatial Information Services (www.tianditu.gov.cn).

The residuals are the values of differences between the observed and predicted values of seropositive rates, which are important key indicators of model structure and performance. The residuals overall demonstrated a random pattern of spatial distribution (Fig 4). The absolute values of the residuals were less than 2.5 and 96.2% (1649/1714) of the areas had residuals between 1 and -1. All of these indicated reliable structure of MGWR in this study [34,46].

## 4. Discussion

This study investigated determinants for the seropositive rate of schistosomiasis in 1714 villages or communities of Human Province and explored their spatial distributions by six spatial regression models. We examined the correlations between the study factors and the seropositive rate at the village level and further explored spatial variation in local space. To the best of our knowledge, it is the comprehensive inclusion of factors, with particular inclusion of interventions such as drinking water sources and toilet conversion at the village level.

In all the models, the nearest distance from a river showed a consistent negative correlation, which was consistent with similar regression coefficients across villages. This negative correlation could be explained by the convenience of exposure to epidemic water for residents [47]. Alene et al. revealed a similar negative association between the distance to water bodies and the schistosome infection rate diagnosed by PCR using a Bayesian spatial model [17]. A deeper analysis of spatial heterogeneity revealed no matter in which village or community, the regression coefficients for this determinant in MGWR were similar and high-contributing. These results in local space further supported the important impact of distance from river on the transmission of schistosomiasis. The proportion of households using well water also showed a negative correlation with the seropositive rate in all models, indicating that using well water played a protective role in the transmission of schistosomiasis. However, there was a spatial heterogeneity for using well water. Although the regression coefficients were negative in the vast majority of areas (Fig 2), a small number of areas surrounding the Dongting Lake demonstrated a positive correlation. One possible reason is that the well water in these areas is contaminated by Dongting Lake, which needs further investigations to identify. Another possible reason is that the local R^2^ in these areas is not high, reducing the explanatory power of positive regression coefficients [48]. For the two determinants, changing the exposure of the contaminated river seems to be the key. Additional measures should be taken to minimize the exposure to infected water, especially for households living near the river.

There was a negative correlation between annual average daytime surface temperature and seropositive rates of schistosomiasis in all villages or communities. Similarly, Su et al. found the daytime land surface temperature was significantly negatively associated with schistosomiasis prevalence by the fixed rank kriging (FRK) model and the integrodifferential equation (IDE) model [49]. For the negative correlation, a possible explanation is that surface temperatures are not conducive to snail density. Previous studies have shown that land surface temperature was associated negatively with snail density [50,51]. Given the positive role of snails in schistosomiasis transmission, surface temperature may reduce schistosomiasis infection by reducing snail density. Notably, the correlations among surface temperature, snail population and schistosomiasis infection vary in different studies, for example, unrelated, positively correlated, negatively correlated or complex correlations [49,52–55]. It may suggest that the correlation between surface temperature and schistosomiasis infection needs to be treated specifically in the context of the actual study area.

The proportion of unmodified toilets had the highest mean positive correlation and was significant in all local areas in MGWR. Previous studies found that toilet renovation could reduce the prevalence of schistosomiasis in both rural and urban areas [56,57]. When the spatial heterogeneity of toilets was further considered in this study, we found toilet renovation was still applicable in all local areas at the village level. Although we did not find detailed data pertaining to toilets, Phillips et al. emphasize the importance of good maintenance and high hygienic quality of toilets in reducing the transmission of schistosomiasis [58].

Some determinants showed a heterogeneity in association with the seropositive rate in different local spaces. For instance, cattle displayed a positive correlation distribution for villages close to Dongting Lake; however, in nearly half of the areas away from the lake, the impact of cattle was insignificant (Fig 2). It laterally supported the effectiveness of interventions targeting cattle in areas around Dongting Lake [59,60]. A spatial variation was also observed for snails that had a strong correlation with the seropositive rate exited in the northwest of Dongting Lake. It may be due to the high density of living snails in these areas [61], where snail control measures should be strengthened.

Due to the impact of various bandwidths and high-order multiple inferences, the distribution of a determinant varies across the local models [62,63]. Compared to GWR, MGWR is often more cautious and effective [23,43], and in this study, the fitted R^2^ increased from 0.792 to 0.821 (Table 4). MGWR also has an advantage of allowing different spatial scales and match varying bandwidth [40].

The study has some limitations. IHA cannot distinguish between recent and distant infections for schistosomiasis [19,64]. Serological data have been frequently used in previous studies due to their high sensitivity [11,19,65], but there is a concern for their low specificity (i.e., high false positive rate) [64,66]. Theoretically, the false positive rate comes from cross-reactions to other parasites than schistosome in the IHA technique and long-term antibody concentration level after infection and treatment [67,68]. The presence of false positive rates means that the seropositive rate amplifies the actual schistosomiasis infection to some extent. Especially in the villages which had reached the transmission interruption or elimination in this study, a certain number of cured schistosomiasis patients resulted in a relatively high false positive rate, which somewhat amplified the regression coefficient of the determinants in models. In addition, this study only analyzed determinants at the village level and lacked the analysis of individual factors. Also, the regression coefficients of determinants differed in spatial models, which might limit the applicability of these findings to some extent.

In this study, MGWR performed better than other models for the spatial variability of schistosomiasis in Hunan province. The spatial variability was different for different determinants. The findings for the determinants for the seropositive rate and mapped variability for some key determinants at the village level can be used for developing precision intervention measure for schistosomiasis control.

## Supporting information

S1 Table All determinants we collected and analyzed initially. (CSV)
